# Contribution of Lung Macrophages to the Inflammatory Responses Induced by Exposure to Air Pollutants

**DOI:** 10.1155/2013/619523

**Published:** 2013-08-22

**Authors:** Kunihiko Hiraiwa, Stephan F. van Eeden

**Affiliations:** Department of Medicine, UBC James Hogg Research Centre, St. Paul's Hospital, University of British Columbia, Vancouver, BC, Canada V6Z 1Y6

## Abstract

Large population cohort studies have indicated an association between exposure to particulate matter and cardiopulmonary morbidity and mortality. The inhalation of toxic environmental particles and gases impacts the innate and adaptive defense systems of the lung. Lung macrophages play a critically important role in the recognition and processing of any inhaled foreign material such as pathogens or particulate matter. Alveolar macrophages and lung epithelial cells are the predominant cells that process and remove inhaled particulate matter from the lung. Cooperatively, they produce proinflammatory mediators when exposed to atmospheric particles. These mediators produce integrated local (lung, controlled predominantly by epithelial cells) and systemic (bone marrow and vascular system, controlled predominantly by macrophages) inflammatory responses. The systemic response results in an increase in the release of leukocytes from the bone marrow and an increased production of acute phase proteins from the liver, with both factors impacting blood vessels and leading to destabilization of existing atherosclerotic plaques. This review focuses on lung macrophages and their role in orchestrating the inflammatory responses induced by exposure to air pollutants.

## 1. Introduction

The inhalation of toxic environmental particles is a worldwide public health problem. There are numerous sources of suspended particulate matter (PM) including industrial sources, automobile traffic, natural disasters, such as forest fires and volcanic eruptions, and local sources generated either in the home or workplace [1–3]. Urban air pollution originates from a variety of sources, of which the combustion of fossil fuel products is the principal source. Air pollutants can be classified by their source, chemical composition, size, mode of release (gaseous or particulate), and space (indoor or outdoor) [4]. Epidemiological studies show that air pollution exposure positively correlates with admissions for pneumonia, asthma, and chronic obstructive pulmonary disease (COPD) [5]. Of all the pollutants, inhalable particles (PM_10_) showed the strongest association with adverse respiratory health effects [6]. In addition, data from large population cohorts have indicated an association between exposure to PM and cardiovascular morbidity and mortality [7–10]. Mechanistically, this is thought to be due to the systemic inflammatory response induced by exposure to PM air pollution [11]. This concept is supported by studies showing a positive association between long-term PM exposure and hematological markers of inflammation and diseases such as diabetes mellitus [12].

Inhalation of air pollution particles induces a local response in the lung that is initiated by alveolar macrophages (AMs) and airway epithelial cells. The macrophages are several times more potent in producing proinflammatory mediators that contribute to the local inflammatory response in the lung but also contribute to the subsequent systemic inflammatory response [11]. The systemic inflammatory response is characterized by mobilization of inflammatory cells from the bone marrow into the circulatory system, followed by their activation, as well as the production of acute phase proteins by the liver, and an increase in circulating inflammatory mediators [13]. In this review, we focus on the role of AMs in inflammation induced by air pollution.

## 2. Response of Lung Macrophages to Inhaled Air Pollutants

### 2.1. Processing of Particulate Matter by Macrophages

The primary role of AM is to keep the air spaces clear by removal of all foreign materials via phagocytosis. Experiments from our laboratory have shown that AMs exposed to atmospheric particulate matter are able to phagocytose these particles *in vivo* and *in vitro* [13, 14]. Nonbiological particles lack specific opsonins preventing them from classic opsonin-dependent phagocytosis. Despite the absence of specific opsonins, AM can phagocytose unopsonized environmental particles [15]. Kobzik identified a role for scavenger-type receptors in this process [16]. The class A scavenger receptor (SR-A) and the macrophage receptor with collagenous structure (MARCO) are considered to be the two major receptors for unopsonized particle phagocytosis by AM [17, 18], and a deficiency in scavenger receptor function results in reduced uptake of environmental particles by AM [18].

Toll-like receptors (TLRs) are sensors that directly recognize molecules from microbes. They are essential for initiation of the innate immune response, when interacting with PM, and also play a role in sustaining and regulating the adaptive immune response to PM. Ambient PM contains small amounts of microbial materials such as lipopolysaccharide (LPS)/endotoxin [19, 20], beta-glucan, bacteria, and fungal spores [21] that are thought to be the mechanism by which TLRs engage in processing PM. Among the TLRs identified in humans, TLR4 and TLR2 are thought to be the two main receptors that bind PM [22]. Toll-like receptor 4 initiates a signaling cascade in response to LPS present in the outer membrane of gram-negative bacteria, while TLR2 initiates signals in response to zymosan (beta-glucans) and peptidoglycan of gram-positive bacteria. Furthermore, microorganisms attached to PM can be opsonized by specific opsonins (such as immunoglobulin Fc receptors and complement receptor 3) that allow AM to phagocytose the particles via an opsonin-dependent pathway [23].

### 2.2. Macrophage Responses to Particle Size and Chemical Composition

Particulate matter is classified according to aerodynamic diameter into PM_10_ (coarse particles, median aerodynamic diameter 2.5–10 *μ*m PM_2.5_ (fine particles, median aerodynamic diameter < 2.5 *μ*m), and ultrafine particles (UFP) median aerodynamic diameter < 0.1 *μ*m). PM_10_ particles are derived predominantly from abraded soil, road dust, construction debris, and oil combustion products with bioaerosols such as fungi, bacteria, endotoxins, and pollen, while PM_2.5_ and UFP are primarily derived from direct emissions from combustion processes such as vehicle use of fossil fuel products, wood burning, and coal burning [4]. Although a considerable amount of data implicate PM_10_ and PM_2.5_ in adverse health effects [24–26], much less is known about the risks of UFP. In addition, several studies have shown that PM_2.5_ and UFP have the strongest association with adverse cardiovascular adverse effects [27, 28], which is a direct consequence of the systemic response induced by these particles [29, 30]. Alveolar macrophages exposed to smaller PM that have the ability to penetrate deep into the lungs significantly contribute to the systemic inflammatory response. Upon contact with particulate pollutants, AMs are activated, produce proinflammatory cytokines, and undergo apoptosis [31]. The capability of inducing apoptosis and inflammation varies with different particle size and concentration [31]. *In vitro* studies have shown that macrophages do recognize the size and shape of their target pathogens [32]; therefore, their response against various particle sizes may be different. It is generally thought that the larger surface area of PM_2.5_ and UFP per unit concentration of PM allows more opportunity for cellular interaction and a downstream biological response. On the contrary, *in vitro* studies comparing the effects of the coarse and fine fraction of PM_10_ showed stronger proinflammatory effects for the coarse particles [33, 34]. 

In addition to PM size and concentration, particle composition has also been reported to impact PM toxicity [35]. The toxicity of PM may stem from their metal content, adhered organic compounds, or other biological components such as LPS. Schins et al. showed that coarse PM induced a greater inflammatory response than fine PM in rats and suggested that, in these larger particles, toxicity is due more to their biological components, such as endotoxin, than their metal content [35]. In other studies, no apparent relationship could be established between pulmonary injury and the concentration of ambient particles or their elemental components such as sulfate (S), zinc (Zn), manganese (Mn), iron (Fe), and copper (Cu) [36]. Diesel exhaust particles (DEPs) without their organic constituents were no longer able to induce apoptosis or generate reactive oxygen radicals in murine and human macrophages *in vitro* [37]. Diesel exhaust particle is a major component of urban PM_10_ pollutants, which comprise 40% of total PM_10_ levels in Los Angeles [38]. The organic extracts were, however, able to induce apoptosis [37]. The water-soluble fraction of pollutant particles and individual soluble metals such as vanadium (V), nickel (Ni), and Fe did not induce apoptosis in human AMs [31]. In addition to these reports that emphasize the toxicity of organic components in PM, nonorganic components such as metals have also been implicated in the pathogenesis of particulate-induced pulmonary inflammation [39]. Vanadium (V), bromine (Br), lead (Pb), and organic carbon had a strong association with pulmonary inflammation [40]. Stone particles of varying composition (mylonite, gabbro, feldspar, basalt, and quartz) induced different cytokine responses in rat AMs [41]. Because ambient particles contain many other nonleachable and leachable components, further studies are needed to identify the toxicity of different particle components.

### 2.3. Macrophage Responses to Other Ambient Chemicals Such As Ozone

In addition to PM, gaseous pollutants such as ozone also have inflammatory effects on the respiratory tract and AM [42–45]. Ozone exposure induces the release of cytokines and fibronectin by AM [46], increases AM recruitment into asthmatic airways [47], and increases the eosinophilic airway response [48]. Nitrogen dioxide (NO_2_) is a precursor to photochemical smog, and its major effect on health as an outdoor pollutant is likely through the formation of ozone. Recent epidemiologic studies conducted worldwide have provided valuable insight into the associations between sulfur dioxide (SO_2_), NO_2_, and carbon monoxide (CO) exposure and increases in cardiopulmonary mortality, such as respiratory and cardiovascular hospital admissions, emergency admissions caused by stroke (NO_2_), and myocardial infarction (NO_2_ and CO) [49–51]. In these studies, NO_2_ inhibited AMs from playing an immunosuppressive role [52]. Alveolar macrophage phagocytosis was significantly suppressed following coexposure of fine carbon particles and SO_2_ [53]. Because air pollution contains both, the contribution of these gaseous pollutants in modulating the response of the AM to PM is complex, poorly understood, and still an area of active investigation.

### 2.4. Mediators Produced When Macrophages Are Exposed to PM

**Figure 1:**
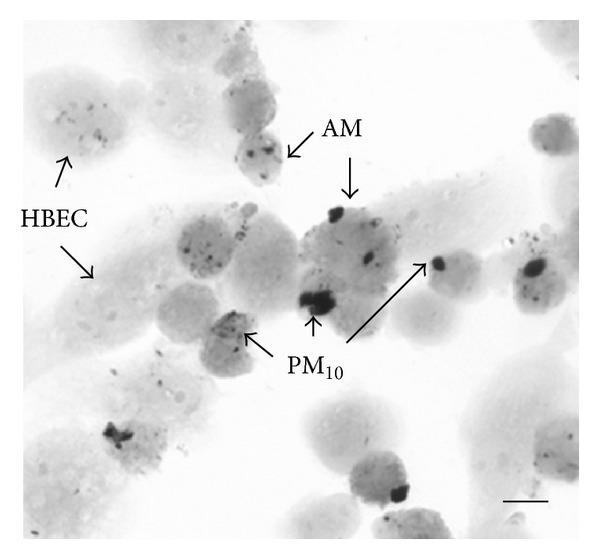
Photomicrograph of cocultured primary human bronchial epithelial cells (HBECs) and human AMs incubated with 100 *μ*g/mL of PM_10_ (EHC-93) for 24 h showing particles internalized by both HBECs and AMs. Cells were cocultured on coverslips, and immunocytochemistry was performed using mouse anti-human CD68 monoclonal antibody to identify AMs. The *bar* represents 10 *μ*m [54].

**Figure 2:**
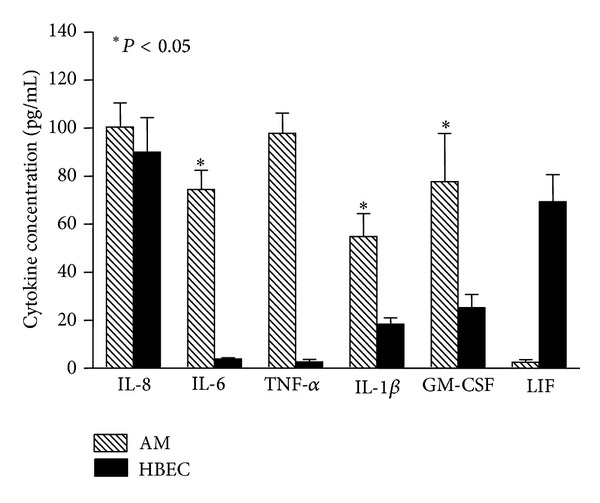
Cytokines produced by human AMs and bronchial epithelial cells (HBECs) when exposed to 100 *μ*g/mL of PM_10_ (EHC-93) for 24 h. Alveolar macrophages produced significantly more IL-6, IL-1*β*, and GM-CSF than bronchial epithelial cells when exposed to same amount of PM_10_.

Alveolar macrophages are one of the most potent producers of proinflammatory mediators in the airways and lung. Studies from our laboratory have shown that human AMs exposed to urban PM (EHC-93) phagocytosed these particles *in vitro* (Figure 1) [13, 54], and, *ex vivo*, they produced tumor necrosis factor alpha (TNF-*α*) in a dose-dependent fashion following PM exposure [14]. In addition, these AMs produce an array of proinflammatory mediators including acute response mediators such as interleukin (IL)-1*β* and IL-6 as well as secondary mediators, such as IL-8, and granulocyte macrophage colony-stimulating factor (GM-CSF) [11, 13]. Interestingly, production of anti-inflammatory mediators such as IL-10 was suppressed [55], suggesting that the AM inflammatory response induced by PM is tipped toward a proinflammatory profile. The inflammatory profile of mediators produced by bronchial epithelial cells exposed to PM is distinct from those of AM [56]. Alveolar macrophages are also more potent producers of the acute response mediators such as IL-1*β*, IL-6, and TNF-*α* than bronchial epithelial cells when exposed to the same dose of PM, suggesting that AMs are the drivers of the proinflammatory response in the lung following inhalation of PM (Figure 2) [56]. Furthermore, instillation of supernatants from human AMs incubated *ex vivo* with urban PM into rabbit lungs produced a systemic response similar to that produced by direct deposition of the same amount of PM directly into the lungs [57, 58], which implies that AMs significantly contribute to the systemic inflammatory response generated following exposure to PM.

### 2.5. Maturation Changes in Macrophages Induced by PM

Macrophages are a heterogeneous population of cells with significant phenotypic plasticity [59]. Depending on the microenvironment, they undergo distinct activation programs, acquiring polarized phenotypes and different functional capacities that together provide an armamentarium that protects, repairs, and sometimes damages tissues [60, 61]. Macrophage “M1 polarization,” also referred to as the “classical activation” program, is induced by signals generated during Th1-mediated immune responses such as interferon (IFN)-*γ* and by exposure to components of pathogens such as bacteria [60, 61]. The M1 polarization response is characterized by upregulation of genes relevant to inflammation and cell-mediated immunity. In contrast, macrophage “M2 polarization” induced upon exposure to the Th2 cytokines IL-4 and IL-13 (referred to as “alternative activation”) or immunoregulatory signals such as IL-10 (also called “deactivation”) is highlighted by induced expression of receptors with scavenger functions, anti-inflammatory cytokines, and molecules implicated in tissue remodeling [60, 61].

**Figure 3:**
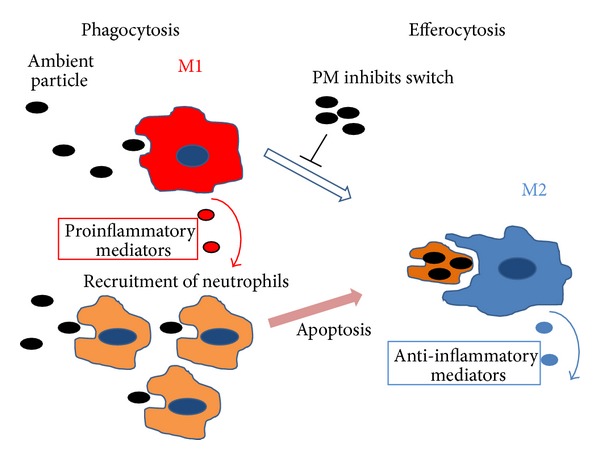
The impact of PM exposure on alveolar macrophage phenotype. PM exposure stimulates macrophages (predominantly M1 phenotype) to produce proinflammatory mediators that attract other immune cells such as neutrophils into the airspaces. Following phagocytosis of the PM, these cells undergo apoptosis and are removed by M2 macrophages, which also produce anti-inflammatory mediators that are pivotal for resolution of the inflammatory response induced by the PM exposure. Persistent inflammation in the lung induced by PM exposure may be due to PM that blocks macrophage switching (M1 to M2), compromising efferocytosis.

It has been reported that cigarette smoke skews the AMs to M2-polarized phenotypes [62]. Although PM shares many of the same ingredients and characteristics as cigarette smoke, the M1 cytokines (IL-12 and IFN-*γ*) are increased consistently in bronchoalveolar lavage fluid (BALF) from PM-exposed animals [63, 64] while the M2 cytokines (IL-4, IL-10, and IL-13) remain at lower levels [65]. Our group previously showed that primary cultured human AM, stimulated *in vitro* with urban PM_10_, produced an array of cytokines without significantly increased levels of IL-10 compared to nonstimulated AM [13]. These reports suggest that PM skews the AMs to an M1 profile rather than an M2 profile. Data from an influenza virus pneumonia model suggest that macrophage pro- and anti-inflammatory phenotypes are under tight control of nearby airway epithelial cells [66]. Epithelial-macrophage crosstalk seems to be an important mechanism in keeping the balance between efficient host defense and excessive inflammation and injury during infection. These responses (M1/M2 switching) of macrophages following PM exposure still need further investigation to assess what factors (such as size or composition) determine switching.  Figure 3 shows how PM exposure could potentially inhibit macrophage switching and thus promote a proinflammatory state.

### 2.6. Macrophage Apoptosis, Autophagy, and Efferocytosis Induced by PM

Alveolar macrophages play numerous roles in immunity, inflammation, and tissue repair. In addition to being key players in the innate immune response against microorganisms and in the initiation of adaptive immune responses, they are crucial for the clearance and processing of microorganisms, dead cells, and environmental debris in the lung tissue via phagocytosis. In contrast to cells such as neutrophils, AMs are long-lived [67] and in general are resistant to apoptotic stimuli [68]. Following activation of AM, by exposure to PM, for example, they either remain in the lung airways or tissues [69] or are removed via the lymphatic system to regional lymph nodes [70]. Several studies have demonstrated that exposure to ambient PM and diesel exhaust particles induced apoptosis in macrophages [37, 71]. Particulate matter-induced apoptosis is considered to be mediated through scavenger receptors [71]. Phagocytosis of apoptotic cells (efferocytosis) by AMs is involved in the regulation of the inflammatory response and maintenance of lung homeostasis by removing dead cells before the onset of necrosis [72]. Alveolar macrophages are primarily responsible for removing and processing dead cells and debris in the airways, thereby reducing their inflammatory potential. Whether air pollution exposure alters the efferocytotic function of AMs is unclear. Our group recently showed that 3-hydoxy-3-methylglutaryl coenzyme A (HMG-CoA) reductase inhibitors (statins) enhance the phagocytic activity of AMs and promote the clearance of PM from lung tissues [73]. Promoting macrophage phagocytosis and efferocytosis could accelerate processing and clearance of PM particles from lung tissues and thereby reduce lung inflammation.

Autophagy consists of the fusion of autophagosomes with lysosomes, forming autolysosomes and resulting in the breakdown of encapsulated materials to components that are then available for homeostasis. Intracellular nanoparticles may undergo autophagic sequestration, and autophagy dysfunction may play an important role in nanoparticle toxicity [74]. Monick et al. identified an autophagic defect in the AMs of smokers and concluded that the decrease in the process of autophagy leads to impaired protein aggregate clearance, dysfunctional mitochondria, and defective delivery of bacteria to lysosomes [75]. Exposure to ambient PM could decrease autophagy of AM in a similar manner to smoking, but further studies are necessary to confirm this.

### 2.7. Interaction of Macrophages with Other Lung Cells

Alveolar macrophages form the first line of defense following inhalation of PM. They sense, scavenge, and phagocytose PM and in the process they produce and release early response cytokines [67]. These cytokines stimulate neighboring airway and alveolar epithelial cells as well as tissue-resident macrophages in an auto- and paracrine manner to produce a variety of chemokines necessary to recruit other cells, such as polymorphonuclear leukocytes, to assist in processing and ultimately clearing foreign material. Human airway and alveolar epithelial cells are also capable of PM endocytosis [54] and in the process they produce mediators such as GM-CSF, IL-1*β*, IL-8, and leukemia inhibitory factor (LIF) in a dose-dependent manner at both the mRNA and protein level when exposed to ambient particles [76]. Coculture experiments of bronchial epithelial cells and AM showed synergistic production of certain mediators such as IL-1*β*, IL-6, and GM-CSF [54]. The increased IL-1*β* production [54, 76] is mediated by the nucleotide-binding domain and leucine-rich repeat protein 3 (NLRP3) inflammasome [77] that spreads the local inflammatory response by interacting with resident dendritic cells residing within or near the epithelium, initiating, and maintaining an adaptive immune response [78]. These studies demonstrate the importance of the interaction of AMs with other lung cells in producing lung inflammation and possibly contributing to the systemic inflammatory response following PM inhalation.

### 2.8. Adaptive Responses Induced by Macrophages Exposed to PM

Alveolar macrophages are also important antigen-presenting cells. After phagocytosis and internalization of PM, the organic components are digested by the endosome into peptide fragments that combine with the MHC class II complex for presentation to CD4+ T cells, which are pivotal steps in cell-mediated and adaptive immunity. Expression of MHC in AMs moderately increased in response to PM exposure in healthy human subjects [79]. Pretreatment of PM with heat to degrade the organic component abolished the MHC class II overexpression, which suggests that the organic component of PM is responsible for MHC class II upregulation [79]. In addition to antigen-specific MHC and T-cell receptor interaction, T cells require a costimulatory signal to be fully activated. These molecules are expressed on the cell membrane of antigen presenting cells and are upregulated by PM exposure [80]. Together, these studies illustrate the crucial role that AMs play in initiating the adaptive immune response to PM exposure. However, the role of this adaptive response in the local lung and systemic inflammatory responses induced by PM exposure has not been well studied to date.

## 3. Macrophages and the Systemic Response Induced by PM

### 3.1. Macrophages and the Bone Marrow Responses

**Figure 4:**
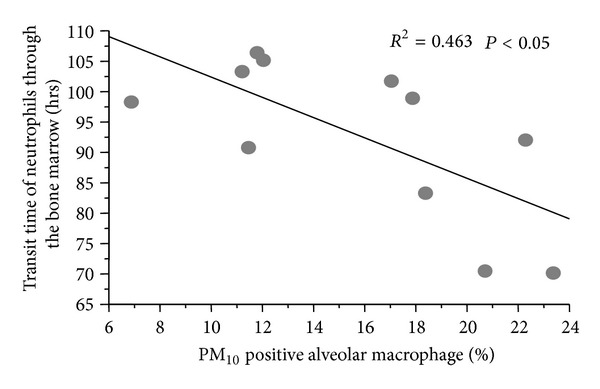
Relationship between the fraction of AMs that phagocytosed PM_10_ particles and the transit time of PMN though the bone marrow. Rabbits were exposed to 5 mg PM_10_ (EHC-93) twice a week for 4 weeks, and AMs with particles in their cytoplasm were enumerated using quantitative histological methods. Dividing PMNs in the marrow were labeled with 5-bromo-2-deoxyuridine and the transit time of PMN through the bone marrow was measured. Faster transit times of PMN through the marrow were associated with more AMs with phagocytosed particles (*R* ^2^ = 0.46, *P* < 0.05) [56].

Earlier studies implicate AMs as key effector cells responsible for generating the systemic inflammatory response associated with exposure to air pollution [13, 41, 56, 81, 82]. They produce a broad range of mediators, particularly IL-6, IL-1*β*, MIP-1*α*, and the hematopoietic growth factor GM-CSF, when exposed to urban PM [13]. The importance of the AM producing the mediators that elicit the systemic response is supported by studies showing a correlation between the amount of particles phagocytosed by AM in the lung and the magnitude of bone marrow stimulation following PM exposure (Figure 4) [56].

Humans exposed to an acute episode of air pollution, where the predominant pollutant was PM, showed increased levels of circulating cytokines such as IL-1*β* and IL-6 and signs of bone marrow stimulation reflected by an increase in circulating band cells counts [83]. These cytokines are similar to those produced by AM exposed to PM both *ex vivo* and *in vivo* [13, 84], suggesting that these mediators produced in the lung enter the circulation and contribute to the systemic response associated with exposure to PM. Recent studies from our laboratory showed that mediators such as IL-6, produced in the lung from PM exposure directly, translocate into the circulation [84] and, because the AMs are the most prolific producer of these mediators following PM exposure, it is reasonable to postulate that AMs are crucially important effector cells in generating the systemic inflammatory response induced by air pollution exposure. 

Several studies from our laboratory have shown that exposure to air pollution stimulates the bone marrow in humans [83] and in animal models [11, 14, 56–58, 85] and promotes the release of both polymorphonuclear leukocytes and monocytes from the marrow. The cytokine production by macrophages in the lung is of particular importance in inducing this systemic inflammatory response; for example, GM-CSF is a hematopoietic growth factor that stimulates granulocyte and monocyte differentiation and releases from the bone marrow, but it also activates circulating leukocytes and prolongs their survival in the circulation [13]. Furthermore, IL-1*β* is one of the “acute response” cytokines that induces cytokine production by many cells, stimulates hematopoiesis, activates endothelial cells, is pyrogenic, and induces the acute-phase response [13]. In addition, stimulation of liver hepatocytes by IL-6 produces acute phase proteins including C-reactive protein (CRP), fibrinogen, and antiproteases [13]. Moreover, IL-6 also stimulates hematopoiesis, specifically the production of platelets, and has a broad stimulating effect on B and T cells, as well as markedly accelerating the transit time of granulocytes through the bone marrow, releasing them into the circulation, and promoting their sequestration in microvascular beds [86]. Collectively, GM-CSF, IL-1*β*, and IL-6 have the ability to elicit a systemic inflammatory response characterized by an increase in circulating leukocytes and platelets by directly stimulating the bone marrow. This bone marrow response elicited by PM exposure is thought to play a critically important role in the downstream adverse systemic health effects associated with exposure to air pollution, particularly the adverse effects on the heart and blood vessels [87]. 

### 3.2. Macrophages and the Vascular Effects of PM

Numerous studies have shown an association between air pollution and increased cardiovascular morbidity and mortality [24, 88–91]. The adverse cardiovascular health effects include hospital admissions and death from conditions such as acute myocardial infarction (acute coronary syndrome), arrhythmias, and congestive heart failure. These hospital admissions were shown to occur within hours of a spike in air pollution exposure. Elevated concentration of PM_2.5_ increased acute myocardial infarction within a few hours [92], which was confirmed by subsequent studies [93]. Seaton et al. proposed that an increase in blood coagulability induced by deposition of particles in the lung is associated with an increase in cardiovascular deaths in susceptible individuals [94]. The systemic response induced by PM_10_ is characterized by activation of the acute-phase response, an increase in coagulation, the release of inflammatory mediators into the circulation leading to activation of the endothelium, and stimulation of the bone marrow causing the release of leukocytes and platelets. These events may contribute to destabilization of atherosclerotic plaques, making them vulnerable for rupture and thrombosis and accounting for the increase in cardiovascular events associated with episodes of air pollution [92].

**Figure 5:**
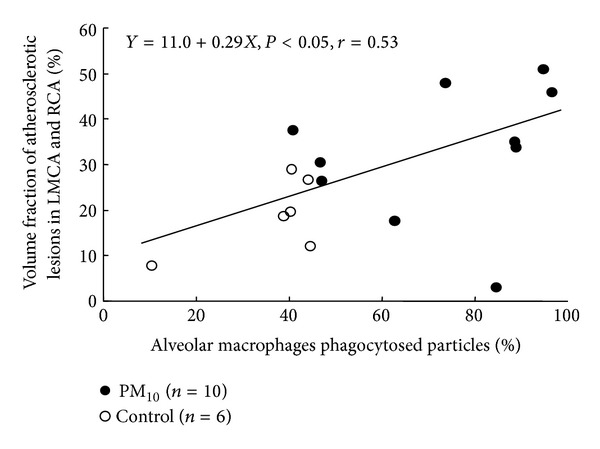
The correlation between the percentage of AMs that phagocytosed particles in the lung and the vol/vol (volume fraction) of atherosclerotic lesions. Results were from rabbits exposed to PM_10_ for four weeks (solid circles; *n* = 10) or saline (control; open circles; *n* = 6) [95].

Mediators such as GM-CSF, IL-1*β*, and IL-6 produced by lung macrophages when exposed to ambient PM have the ability to elicit a systemic inflammatory response characterized by an increase in circulating leukocytes, platelets, and proinflammatory and prothrombotic proteins. These mediators also have the ability to activate circulating leukocytes and the endothelium of the vascular bed to promote leukocyte-endothelial adhesion and migration, contributing to atherosclerotic plaque activation and instability. Our group showed that 4 weeks of exposure to ambient particles in Watanabe hereditarily hyperlipidemic rabbits induced a systemic inflammatory response that included stimulation of the marrow and caused progression of atherosclerosis in both the aorta and coronary arteries, with phenotypic changes in atherosclerotic plaques characteristic of plaque vulnerability [95]. These observations have recently been confirmed by others using ambient PM_2.5_ [96] and by our group, using DEPs in a mouse model [87]. To determine the role of AMs in these effects, studies by our group examined the relationship between the fraction of macrophages in the lung that had phagocytosed particles and circulating mediators strongly associated with cardiovascular disease in humans such as IL-6 [97] and found a positive association [84, 98]. In addition, our group showed an association between the extent of progression of atherosclerosis and features of plaque vulnerability and the fraction of AMs that had phagocytosed PM (Figure 5) [95]. Collectively, these studies strongly implicate the AM in eliciting the systemic inflammatory responses induced by exposure to PM as well as the downstream adverse vascular effects of air pollution.

### 3.3. Interindividual Variability in the Effects of Air Pollutants

The studies mentioned earlier describe the effect of PM on AMs; however, it is understandable that there is marked interindividual variability in the response to PM. Exploration of genetic predispositions and epigenetic changes associated with ambient air pollution shows promising results [99]. Polymorphisms in genes coding for glutathione-S-transferases (GSTs), which are enzymes responsible for the metabolism of reactive oxygen species, are correlated with the risk of lung diseases such as asthma when individuals are exposed to ambient air pollution [100, 101]. Kerkhof et al. showed that single-nucleotide polymorphisms in *TLR2* and *TLR4* genes significantly modified the effect of PM_2.5_ on the incidence of asthma [102]. Ambient air pollution can induce epigenetic changes such as DNA methylation [103–107] and there is further promise for genome-wide association studies (GWAS) [99]. Little is known about the effect of gene-environment interactions on AM and more research in this field is required. 

## 4. Conclusions

**Figure 6:**
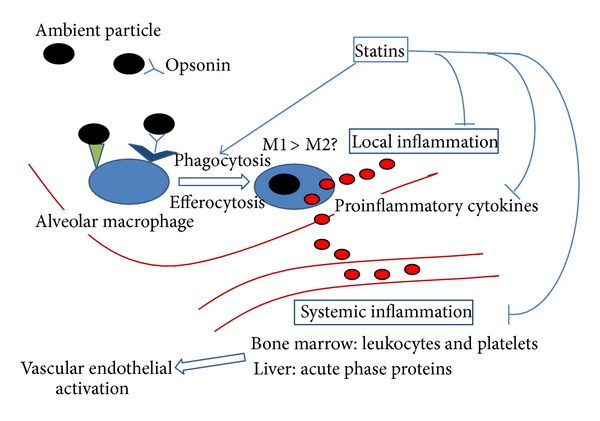
The potential impact of the 3-hydroxy-3-methylglutaryl coenzyme A reductase inhibitors (statins) on both the local (lung) and systemic inflammatory responses induced by exposure to PM. Statins enhance both the opsonized and unopsonized phagocytosis of PM by AMs, potentially promoting the switching of M1 to M2 macrophages (promoting resolution of inflammation), and reduce the production of proinflammatory mediators produced by macrophages when exposed to PM as well as reducing the translocation of these mediators into the systemic circulation. These effects attenuate the systemic inflammatory response induced by PM as well as the downstream adverse vascular effects (endothelial dysfunction and progression of atherosclerosis).

Increasing evidence over the past 10 years has demonstrated that AM plays a key role in local lung and systemic inflammatory responses induced by exposure to ambient PM. In the lung, AM contributes to the magnitude and the nature of the inflammatory response by interacting with other lung cells such as bronchial epithelial cells and dendritic cells in an effort to process and clear the PM from the lung. These macrophages also produce the mediators that are associated with the systemic inflammatory response induced by PM exposure, and recent studies support the concept that these systemic mediators translocate from the lung tissues into the circulation. The adverse systemic health effects of exposure to PM, particularly the adverse cardiovascular effects, are strongly associated with the amount of PM phagocytosed by AMs, underscoring the crucial role that the AMs play in these adverse systemic responses of PM exposure. Therefore, it is reasonable to suppose that attenuating the local and systemic inflammatory responses of AMs, induced by PM exposure, would be of benefit. Recent studies by our group have shown that statins reduce both local lung and systemic inflammatory responses induced by exposure to ambient PM in a rabbit model [73].  Figure 6 shows a potential mechanism by which statins could alter key pathways to achieve these local and systemic anti-inflammatory effects. Downregulating the inflammatory responses could potentially reduce the adverse clinical pulmonary and cardiovascular effects of air pollution. Further research surrounding gene-environment interactions in AMs will contribute to understanding of interindividual variability and may assist in designing tailor-made therapies for air-pollution-related lung and heart diseases.
